# Organogenesis and Ultrastructural Features of *In Vitro* Grown *Canna indica* L.

**DOI:** 10.1155/2016/2820454

**Published:** 2016-01-18

**Authors:** Sharifah Nurashikin Wafa, Rosna Mat Taha, Sadegh Mohajer, Noraini Mahmad, Bakrudeen Ali Ahmed Abdul

**Affiliations:** Institute of Biological Sciences, Faculty of Science, University of Malaya, 50603 Kuala Lumpur, Malaysia

## Abstract

An efficient protocol for micropropagation of* Canna indica *L., an economically and pharmaceutically important plant, was standardized using rhizome explants, excised from two-month-old aseptic seedlings. Complete plant regeneration was induced on MS medium supplemented with 3.0 mg/L BAP plus 1.5 mg/L NAA, which produced the highest number of shoots (73.3 ± 0.5%) and roots (86.7 ± 0.4%) after 2 weeks. Furthermore, the optimum media for multiple shoots regeneration were recorded on MS enriched with 7.0 mg/L BAP (33.0 ± 0.5%). Plantlets obtained were transplanted to pots after two months and acclimatized in the greenhouse, with 75% survival. In addition, ultrastructural studies showed that rhizomes of* in vitro* grown specimens were underdeveloped compared to the* in vivo* specimens, possibly due to the presence of wide spaces. Meanwhile, the leaves of* in vivo* specimens had more open stomata compared to* in vitro* specimens, yet their paracytic stomata structures were similar. Hence, there were no abnormalities or major differences between* in vitro* regenerants and mother plants.

## 1. Introduction

*Canna indica* L. belongs to the family Cannaceae and is a tropical herb grown from rhizomes and seeds, with banana-like leaves and multicolour flowers. The plant can reach a height of 3 meters and their habitat includes shady areas, wet places in forest and savannah, and swampy areas, along rivers or roadsides. Though* C. indica* are grown primarily as an ornamental, a recent study shows that proteins contained in the water extract from* C. indica* fresh rhizome have a potent ability to inhibit human immunodeficiency reverse transcriptase (HIV-RT) virus* in vitro* (93% at 200 *μ*g/mL) [1]. In a different study, leaf samples of* C. indica* exhibited the strongest anti-HIV 1 activity compared to roots and rhizome due to the presence of plastocyanin [2]. Furthermore, many parts of* C. indica* were used in traditional medicine as diaphoretic and diuretic in fevers and dropsy, as a demulcent, to stimulate menstruation, treat suppuration, and rheumatism and to regain energy [3]. It has also been studied for its antitumor, cytotoxic activity and antibacterial properties [4, 5]. Apart from its profound medicinal values, rhizomes of* C. indica, *which are rich in starch, have traditionally been consumed as boiled rhizome and noodles [6] and used to make alcoholic beverages and flour. Meanwhile, the leaf extracts of* C. indica* showed great potential as botanical molluscicide [7] and the flower extract exhibited abilities as a natural indicator in acid-base titration [8].

Despite its many medicinal values and uses, traditional propagation of* C. indica* is hindered by its extremely hard seed coat [9] and slow vegetative propagation of rhizome which is also susceptible to viral infection during multiplication [10–13]. Furthermore, common practice of asexual propagation through rhizome produces genetic stagnancy and genetic variation limitations in* Canna* [14]. Plant tissue culture provides a solution whereby the method rapidly micropropagates plants of superior qualities and free from microorganisms in a relatively short time and minimal space using few starting materials. At the same time, having a reliable micropropagation protocol is a preliminary requirement for any genetic manipulation and protoplast fusion in order to improve* Canna* variety. The first attempt in this regard using intact rhizome of* C. indica* did not show high morphogenetic potential and the plant produced insufficient endogenous cytokinin to induce buds proliferation [15]. Later, the same author tried to stimulate shoot development or bud formation from meristem tips with little success [16]. Recently, a study managed to obtain complete plantlets from leaves explant of* C. indica* via indirect regeneration through* in vitro* callus and somatic embryogenesis [14].

Considering the norm of vegetative propagation for this plant, current study focuses on establishing direct organogenesis and efficient protocol for* in vitro* regeneration system of* C. indica* from rhizome explants. Furthermore, plan for future development and commercialization of this plant requires optimum media for induction of multiple shoots. Lastly, as an early detection system for occurrence of somaclonal variation, ultrastructural features of* in vivo* and* in vitro* samples from leaves and rhizomes of this species were examined using Field Emission Scanning Electron Microscope (FESEM).

## 2. Materials and Methods

### 2.1. Seeds Scarification and Establishment of Sterile Culture

Initially, we attempted culture with leaf and rhizome explants from* in vivo* conditions; however, we encountered high problems of contamination and browning. To overcome these, we grew plantlets aseptically in the laboratory and used different seed scarification methods to increase the germination rate. Seeds of red flowered* C. indica* were purchased from TROPILAB*®*, St. Petersburg, Florida, and germinated under* in vitro* conditions and on garden soil in the green house as control. Aseptic seedlings were obtained by subjecting* C. indica* seeds to chemical scarification of sulphuric acid (H_2_SO_4_) [9]. The process begins with soaking the seeds in distilled water for 1 day to dulcify or soften the seeds. Next, the seeds were soaked in 100% sulphuric acid (H_2_SO_4_) for 3 hours, then rinsed several times, and further soaked in distilled water for one day on a shaker table at 1000 rpm. Prior to inoculation in the laminar flow hood, seeds were surface-sterilized with 70% alcohol for 2 min followed by repeated washing with sterile distilled water. Seeds were then germinated on basal Murashige and Skoog (MS) [17] medium supplemented with 3% (w/v) sucrose and 0.25% gellan gum (Gelzan) as solidification agents [18]. Each flask of 20 mL media was inoculated with one seed and germinated seeds were allowed to grow profusely for two months in culture room at 25 ± 1°C under a 16-hour photoperiod, before the rhizomes were excised as explants.

### 2.2. Complete Plantlet Regeneration and Shoot Multiplication

For organogenesis study, the nutrient medium was prepared by combining commercial MS salts (Sigma), 3% (w/v) sucrose, and 0.25% Gelzan as gelling agent. Plant growth regulators (PGRs) were added to the medium prior to adjustment of pH (5.8) and sterilization (autoclaving at 120°C for 20 min). About 20 mL of the media was evenly dispensed into sterilized tubes where one explant was inoculated per tube. Approximately 5 rhizome explants (~1.0 cm^2^) per aseptic seedlings were excised and cultured in a horizontal position with the cutting side facing the MS medium augmented with combinations of 1.5–3.0 mg/L 6-benzylaminopurine (BAP) with 1.5–4.5 mg/L 2,4-dichlorophenoxyacetic acid (2,4-D), 1.5–3.0 mg/L BAP with 1.5–4.5 mg/L *α*-naphthalene acetic acid (NAA), and 1.5 mg/L kinetin (KN) with 1.5–4.5 mg/L NAA to observe direct organogenesis into complete plantlet. Meanwhile, multiple shoots were tried to induce using single hormone of BAP (0.5–9.0 mg/L), KN (0.5–9.0 mg/L), NAA (0.5–3.0 mg/L), and Triiodobenzoic acid (TIBA; 0.5, 1.0 mg/L). All chemicals used were of analytical grade (Sigma Chemical Co., USA). Each treatment consists of 30 replicates. The cultures were maintained in the culture room at temperature of 25 ± 1°C under a 16-hour photoperiod with a photosynthetic photon flux density of 50 *μ*mol m^−2^ s^−1^ provided by cool white fluorescent lamps.

The cultures were observed daily for any contamination and explants suffering from minor contamination will quickly be transferred into new media. Tubes containing fast growing explants will require frequent subculturing into new media or container in order for growing process to continue. Plant responses were observed and recorded for each replicate after 8 weeks. Multiple shoots induced were later excised and cultured on optimum media for complete plant regeneration.

### 2.3. Acclimatization of Plantlet

After 2 months, complete plantlets with well-developed shoots and roots from media of direct organogenesis were removed from the culture medium, washed gently under running tap water, and transferred to plastic pots containing black (peat) soils under diffuse light conditions [19]. Plantlets were then covered with clear plastic bags (with holes) and placed in the culture room at temperature of 25 ± 1°C for 7 days prior to field transfer to the greenhouse. Watering with tap water was done twice a day with maximum volume of 50 mL per session, at early morning and late evening, for the period of 3 months with constant monitoring.

### 2.4. Statistical Analysis

All data and variables were statistically analyzed using SPSS statistical package version 11 (SPSS Inc., Chicago, III). Values are presented as mean ± SE. One-way ANOVA and Multiple Range Analysis were done on all data using Duncan's test at *p* = 0.05.

### 2.5. Ultrastructural Studies Using Field Emission Scanning Electron Microscope (FESEM)

Fresh rhizomes and leaves (2 months) of* in vivo *and* in vitro C. indica* were collected and cleaned prior to excise into small pieces (specimen) to exhibit important parts of the samples to be viewed. The samples were placed on a stub and examined using a JEOL JSM-7001F Field Emission Scanning Electron Microscope (FESEM).

## 3. Results and Discussion

### 3.1. Establishment of Aseptic Seedlings

Preliminary experiments showed that* C. indica* seeds did not germinate satisfactory in the* in vitro* condition without scarification. Therefore, the black hard seed coat was scarified mechanically and chemically. The best scarification method was soaking of seeds for three hours in sulphuric acid (H_2_SO_4_) which produced the highest percentage of successful seeds germination (84.0%  ± 0.4). Similarly, the positive effects of H_2_SO_4_ scarification (more than 90.0%) were also reported by other researches for* C. indica* seeds sown in the* in vivo *conditions [9]. In regard to these studies, the slight variations in germination percentage between* in vivo* and* in vitro* seed germination could be attributed to the variability of sowing medium and the vigorous method of seeds sterilization. Furthermore, differences in seed viability could be caused by nonhomogeneous seed lots due to uneven maturation and differences in seed dormancy levels [20]. Scarification using H_2_SO_4_ was also favoured by seeds of tamarind [21] and bladder-senna [22]. The coatless seed commences germination by breaking the quiescence or dormancy. Process of seed development begins with the formation of radicle (growing root) after day 5. This is followed by the coleoptile which encloses the embryonic shoots and then pushes upward to the surface after day 15. Both will resume elongation and promoting multiple roots, hairy roots, and rhizome after day 30. Once elongation process stops, the first leaf emerged. After two months, aseptic seedlings with well-developed roots and leaves started to accumulate in the sterile tube. Seedlings were then ready to be used as source of explants.

### 3.2. Induction of Shoots, Roots, and Multiple Shoots

**Figure 1:**
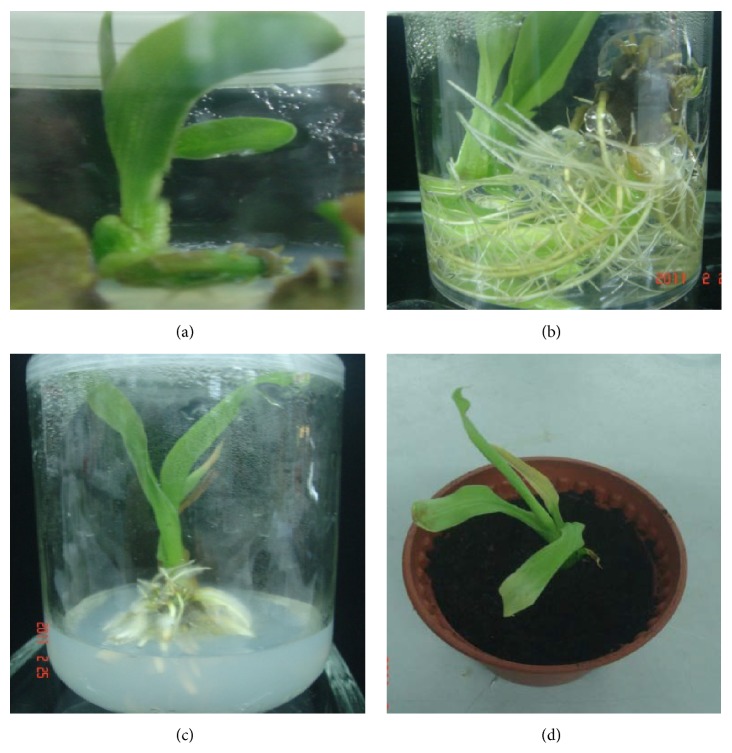
Different stages of* Canna indica *L. plantlet development. (a) Complete plant regeneration after 1 month. (b) Vigorous plantlet growth after 2 months. (c) Subcultured plantlet in a larger container. (d) Acclimatized plantlet on garden soil.

Rhizome explants produced new shoots and roots simultaneously after two weeks of inoculation on MS medium supplemented with combinations of BAP plus NAA. However, root and shoot induction varied in different concentrations of the hormones. High frequency of shoots (73.3%) and roots (86.7%) which resulted in complete plantlet regeneration (Figure 1(a)) was observed in MS medium supplemented with 3.0 mg/L BAP plus 1.5 mg/L NAA. The plantlets grew vigorously (Figure 1(b)) and subsequently required subculturing into larger containers (Figure 1(c)). After 3 months, the plantlets were acclimatized on garden soil, first in the culture room for 7 days and later in the greenhouse (Figure 1(d)).  Table 1 shows that lower concentration of BAP with higher concentration of NAA only induced high formation of roots, while increasing the concentrations of NAA with optimum concentration of BAP reduced both shoot and root formation. Meanwhile, the combination of KN and NAA was not as effective as the other hormone combinations at promoting shoots and roots development. In contrast, a previous study [15] reported that intact rhizome explants produced shoot buds and regenerated into complete plantlets effectively in media containing a combination of 2 mg/L IAA and 1 mg/L KN. A further study by Kromer and Kukulczanka [16] was also in disagreement with the present study, whereby they preferred combinations of KN, adenine sulphate, and NAA to combinations of BAP plus NAA. The combinations of BAP plus NAA are substantially important in propagation of various ornamental species, whereby at equal concentration, the combination can yield production of callus while at other concentrations it can result in direct regeneration and rhizogenesis [17].

The response of rhizome explants to various plant growth regulators used singly, in terms of shoot, root, and multiple shoot formation, is depicted in Table 2. All single hormones tested induced formation of shoots and roots but at different induction levels. According to the table, the percentage of shoot formation increased with the increase of BAP up to the optimum concentration of 5.0 mg/L and later gradually decreased. The pattern was in agreement with previous researchers [14, 23] which observed a less effective formation of shoots with higher concentrations of BAP. The variants in optimum concentration of BAP for regeneration could be attributed to the types of explants used. Regeneration of plantlets from somatic embryos of* C. indica* only required low concentration of BAP (2.0 mg/L) [14] while, in the present study, rhizome explants required a high concentration of BAP (5.0 mg/L) to induce optimum shoot and root formation. Similarly, rhizome buds of* Curcuma manga* give the best response of shoot formation in MS medium containing a high concentration of BAP (9.0 mg/L BAP) [24]. In media with KN only at the concentrations tested, the percentage of shoot formation recorded was less than 60%, while inversely, more than 60% root formation was promoted with the gradual increase of its concentration. A low concentration of TIBA (0.5 mg/L) encouraged formation of shoots, while at higher concentration, the formation of shoots declined drastically and only formation of roots was promoted. TIBA is an antiauxin that promotes lateral shoot initiation in shoot tip culture of* Canna edulis* [18] and indirect regeneration of turmeric from callus [25]. Among the cytokinins used, BAP was found to be the most suitable in promoting cell division, shoot multiplication, and axillary bud formation, while inhibiting root development [26]. However, we observed profuse rooting in all the cases, which may be a general phenomenon for* Canna* [14] and the members of Zingiberales as similar phenomena were previously reported in ginger [27] and turmeric [28].

**Figure 2:**
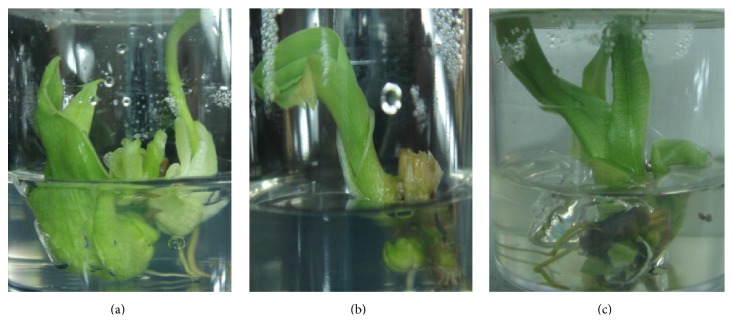
Multiple shoot induction from* Canna indica *L. rhizome explants, in (a) 7.0 mg/L BAP; (b) 6.0 mg/L BAP; and (c) 5.0 mg/L BAP after 8 weeks.

Multiple shoots of* C. indica* with more than 20.0% induction were successfully obtained in MS media supplemented with the single hormone of BAP (3.0, 5.0, 7.0, and 9.0 mg/L) and KN (9.0 mg/L) (Table 2). The multiple shoots were healthy with normal looking leaves and roots. The highest number of multiple shoots formation (33.0% ± 0.48) with occurrence of five multiple shoots per explant (Figure 2(a)) was recorded in MS medium supplemented with 7.0 mg/L BAP. Furthermore, there were also observations of four and three multiple shoots per explant on MS medium supplemented with 5.0 mg/L BAP (Figure 2(b)) and 6.0 mg/L BAP (Figure 2(c)), respectively. Induction of multiple shoots using a combination of cytokinin and auxin has been reported by many researchers [24, 29, 30] and is considered as an important step for commercial exploitation of a micropropagation protocol. However, the ability of producing multiple shoots with roots using a single hormone (cytokinin) in a tissue culture system is highly desirable, especially in reducing costs for mass production of any species. However, the optimum requirement at propagule proliferation stage differs from species to species. In research involving other species related to* C. indica*, shoot multiplication of* C. aromatic* was found to be optimal at 5 mg/L BAP [31] and for* C. zedoary*, 3 mg/L BAP was reported to be suitable for multiplication [32].

Acclimatization of plantlets is the crucial phase where plantlets are in transition from* in vitro* phase to* in vivo* phase. Plantlets with well-developed shoot and roots were removed from the culture media and transferred to plastic pots containing black (peat) soils. They were maintained for about 7 days under plastic covers to avoid desiccation prior to field transfer to the greenhouse. The gradual transition process from culture container to the greenhouse produced normal plant growth and morphology with plant survival rate as high as 75.0% by minimizing physiological stress on the regenerated plantlets [33]. Similarly, in another study* C. indica* acclimatized in clay pots containing an autoclaved mixture of soil and sand produced 72.2% survival rate of regenerated plantlets [14]. On the other hand, a simple acclimatization method was preferred for* Kaempferia galangal,* whereby a survival rate of 80–90% was easily achieved by dismissing the hardening process and simply keeping the plants in 55% shade and watering twice a day [34].

### 3.3. Ultrastructural Studies

**Figure 3:**
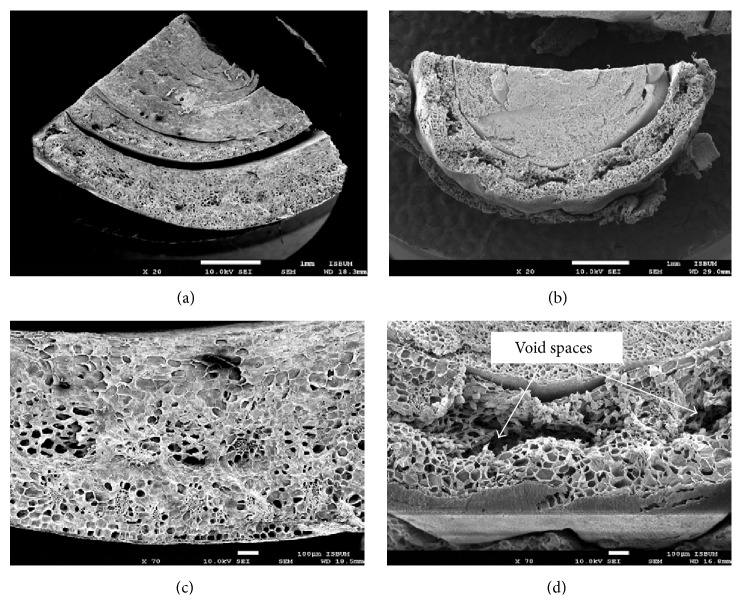
Ultrastructural comparison of* Canna indica *L. rhizome. The outer layer of* in vivo* rhizome (a) is smoother and well-formed compared to the* in vitro* rhizome (b). At 70x magnification, there are no conspicuous void spaces in the* in vivo *rhizome (c) while void spaces were visible in the* in vitro* rhizome (d).

In a tissue culture system, it is important to produce plantlets identical to the parent plants and avoid formation of somaclonal variation. The present study provides the first comparison between rhizome specimens from* in vivo* and* in vitro C. indica* under field emission scanning electron microscope (FESEM). Macromorphologically, no morphological abnormalities in tissue culture raised plants were observed when compared to plants grown in field. However, going deeper into rhizome ultrastructural observations, there were minor structural differences, especially at the outer layer of the rhizome (Figures 3(a) and 3(b)). The* In vivo* specimen (Figure 3(c)) exhibited an absence of void spaces, while void spaces were very apparent in the* in vitro* specimen (Figure 3(d)). This indicated that the* in vitro* rhizome was underdeveloped compared to the* in vivo* rhizome specimen. It could be hypothesized that since the culture medium was providing all the necessary foods and nutrients to the plant, thus the rhizome's natural function as a storage compartment for food materials such as starch was underutilized. Furthermore, rhizomes of specimens grown in* in vitro* conditions required a longer time to reach their full developmental stage or maturity, even though both specimens were collected at the same plant age. According to Mahmad et al. [35],* in vitro *lotus plants (*Nelumbo nucifera *Gaertn.) grew vigorously and possessed similar physiological characteristics as the mother plant only after 8 months of acclimatization.

**Figure 4:**
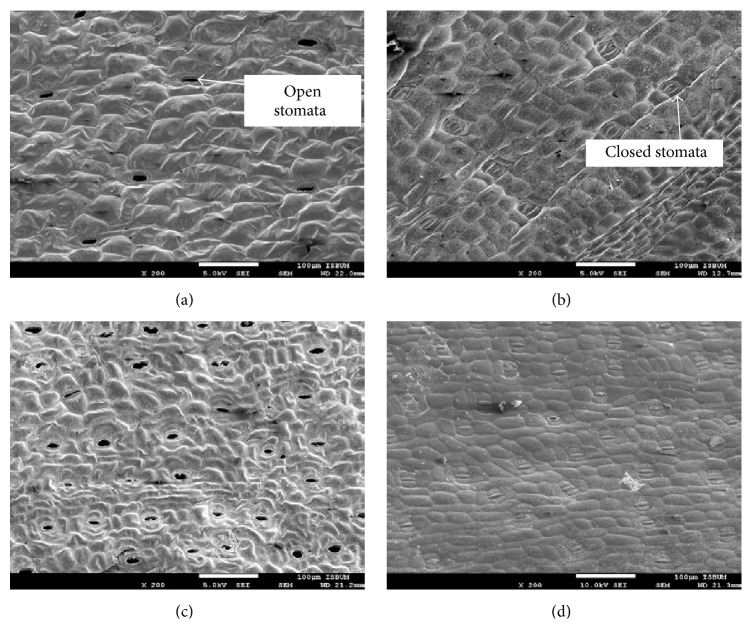
Surface view of stomata distribution of* Canna indica *L. leaves. More open stomata are detected on the* in vivo* leaves ((a) adaxial; (c) abaxial) compared to* in vitro* leaves ((b) adaxial; (d) abaxial).

**Figure 5:**
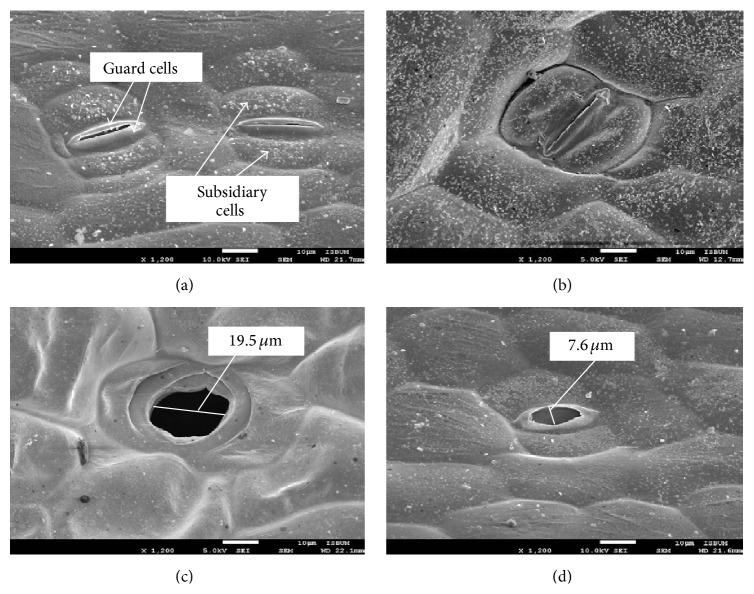
Ultrastructural comparison of stomata structure of* Canna indica *L. leaves. Stomata of* in vivo *(a) and* in vitro* (b) leaves are paracytic in structure and the pores of* in vivo* stomata (c) are larger in comparison with the stomata of* in vitro* leaves (d).

Stomata distribution and structure were compared between adaxial and abaxial leaf surface of both* in vivo* and* in vitro* specimens of* C. indica.* Figure 4(a) shows that there are an abundance of open stomata detected on the leaf surfaces of* in vivo* specimens compared to* in vitro* specimens that are rich in closed stomata (Figure 4(b)). Open stomata distribution was also much more visible on the abaxial surface (Figure 4(c)) compared to the adaxial surface (Figure 4(a)) of* in vivo* leaves. This is a normal and expected occurrence of leaves from* in vivo* conditions. Abundance of closed stomata of* in vitro* leaves on both adaxial (Figure 4(b)) and abaxial (Figure 4(d)) surfaces could be the result of nonfunctional stomata, which was similarly reported in the potato plant [36]. The stomata were probably nonfunctional or failed to mature to a normal functioning state due to prolonged exposure of the plantlets to high relative humidity and low CO_2_ concentration. The presence of sucrose in the medium and accumulated ethylene in the headspace of the vessel might also have played a role [37]. As a consequence, photosynthesis, transpiration, and uptake of water, nutrients, and CO_2_ could be suppressed and dark respiration enhanced, resulting in poor growth [38]. However, functioning closed stomata or plantlet with no fully open stomata is a great acclimatization condition for* in vitro* plantlets whereby, as a consequence, the plantlet will only wilt slightly and the epidermal cells can recover and become turgid quickly after transfer to* ex vitro *conditions, leading to increased survival rate [39]. The surface view shows that both* in vivo* (Figure 5(a)) and* in vitro *(Figure 5(b))* C. indica* leaves possess paracytic stomata, characterized by the position of the subsidiary cell which is parallel to the guard cells [40]. It has typical monocot stomata in which the dumbbell-shape guard cells with unevenly thickened walls are dwarfed by the larger subsidiary cells, which are lacking in dicot stomata [41]. Furthermore, the subsidiary cells and the long axes of the stomata lie parallel to each other. The pores of* in vivo* stomata (Figure 5(c)) are almost three times bigger than the pores of* in vitro* stomata (Figure 5(d)).

Electron microscopical observations of rhizome and leaves belonging to regenerants from* in vitro* culture of* C. indica* present from many points of view a similar structure with those from the native plants, which demonstrates that our experimental* in vitro* conditions did not induce significant morphological alterations at the ultrastructural level. Direct complete plant regeneration of* C. indica* was possible using sterile rhizome explants cultured in MS medium supplemented with 3.0 mg/L BAP plus 1.5 mg/L NAA. Meanwhile, multiple shoots were successfully induced in medium with a high concentration of the single hormone BAP (7.0 mg/L BAP). The finding paves the way for future research on biotechnology manipulations and commercialization, especially in producing virus-free plants and synthetic seed of these medicinally important ornamental species. Furthermore, all plantlets were successfully acclimatized to the greenhouse with 75% survival rate.

## Figures and Tables

**Table 1:** The combined effects of hormones on new shoot and root formation of *Canna indica* L. after 8 weeks.

MS + hormones (mg/L)	New shoot formation (%)	Number of shoots per explant	New root formation (%)	Number of roots per explant
1.5 BAP + 1.5 2,4-D	0.0	0	0.0	0
1.5 BAP + 4.5 2,4-D	0.0	0	0.0	0
3.0 BAP + 1.5 2,4-D	0.0	0	0.0	0
3.0 BAP + 3.0 2,4-D	0.0	0	0.0	0
3.0 BAP + 4.5 2,4-D	0.0	0	0.0	0
1.5 BAP + 1.5 NAA	10.0 ± 0.3^a^	1	53.3 ± 0.5^ab^	6
1.5 BAP + 3.0 NAA	13.3 ± 0.4^a^	1	60.0 ± 0.5^ab^	7
1.5 BAP + 4.5 NAA	23.3 ± 0.4^ab^	1	73.3 ± 0.5^bc^	8
3.0 BAP + 1.5 NAA	73.3 ± 0.5^c^	1	86.7 ± 0.4^c^	10
3.0 BAP + 3.0 NAA	40.0 ± 0.5^b^	1	56.7 ± 0.5^ab^	5
3.0 BAP + 4.5 NAA	43.3 ± 0.5^b^	1	66.7 ± 0.5^abc^	6
1.5 KN + 1.5 NAA	0.0	0	0.0	0
1.5 KN + 3.0 NAA	3.3 ± 0.2^a^	1	43.3 ± 0.5^a^	4
1.5 KN + 4.5 NAA	0.0	0	0.0	0

Data represent mean ± standard error (SE) from 30 replicates per treatment. Means with different letters in the same column are significantly different at *p* = 0.05 according to Duncan's multiple range test (DMRT).

**Table 2:** Effects of single hormone on shoots, multiple shoots, and root formation of *Canna indica* L. after 8 weeks.

MS + hormone (mg/L)		Shoot formation (%)	Multiple shoots formation (%)	Number of shoots per explant	Root formation (%)	Number of roots per explant
MS (control)		26.7 ± 0.5^abc^	0.0	1	30.0 ± 0.5^ab^	5

BAP	0.5	46.7 ± 0.5^bcde^	0.0	1	56.7 ± 0.5^cde^	7
	1.0	56.7 ± 0.5^def^	3.0 ± 0.2^a^	2	73.3 ± 0.5^de^	9
	2.0	46.7 ± 0.5^bcde^	3.0 ± 0.2^a^	2	50.0 ± 0.5^abcd^	7
	3.0	70.0 ± 0.5^ef^	20.0 ± 0.4^abcd^	3	56.7 ± 0.5^cde^	8
	5.0	76.7 ± 0.4^f^	30.0 ± 0.5^cd^	4	53.3 ± 0.5^bcde^	6
	7.0	70.0 ± 0.5^ef^	33.0 ± 0.5^d^	4	36.7 ± 0.5^abc^	4
	9.0	53.3 ± 0.5^cdef^	30.0 ± 0.5^cd^	4	26.7 ± 0.5^a^	3

KN	0.5	46.7 ± 0.5^bcde^	3.3 ± 0.2^a^	2	60.0 ± 0.5^cde^	8
	1.0	43.3 ± 0.5^abcde^	0.0	1	66.7 ± 0.5^de^	9
	2.0	36.7 ± 0.5^abcd^	6.7 ± 0.3^a^	2	70.7 ± 0.4^ef^	10
	3.0	33.3 ± 0.5^abcd^	3.3 ± 0.2^a^	2	66.7 ± 0.5^de^	7
	5.0	56.7 ± 0.5^def^	6.7 ± 0.3^a^	2	73.3 ± 0.5^de^	8
	9.0	56.7 ± 0.5^def^	26.7 ± 0.5^bcd^	3	70.0 ± 0.5^de^	7

NAA	0.5	26.7 ± 0.5^abc^	0.0	1	100.0 ± 0.0^f^	12
	1.0	23.3 ± 0.4^ab^	10.0 ± 0.3^ab^	2	100.0 ± 0.0^f^	13
	2.0	20.0 ± 0.4^ab^	3.3 ± 0.2^a^	2	100.0 ± 0.0^f^	12
	3.0	16.7 ± 0.4^a^	0.0	1	100.0 ± 0.0^f^	14

TIBA	0.5	70.0 ± 0.5^ef^	13.3 ± 0.4^abc^	2	50.0 ± 0.5^abcd^	6
	1.0	33.3 ± 0.5^abcd^	3.3 ± 0.2^a^	2	70.0 ± 0.5^de^	10

Data in percentage are mean ± standard error (SE) from 30 replicates per treatment. Means with different letters in the same column are significantly different at *p* = 0.05 according to Duncan's multiple range test (DMRT).
